# Retraction Note: Upregulation of hsa_circ_0002003 promotes hepatocellular carcinoma progression

**DOI:** 10.1186/s12885-024-12891-6

**Published:** 2024-09-09

**Authors:** Lisha Zhou, Qianwen Wang, Jun Hou, Xiangwei Wu, Lianghai Wang, Xueling Chen

**Affiliations:** 1 NHC Key Laboratory of Prevention and Treatment of Central Asia High Incidence Diseases, First Affiliated Hospital, Shihezi University School of Medicine, Shihezi, Xinjiang China; 2 Key Laboratory of Xinjiang Endemic and Ethnic Diseases, Shihezi University School of Medicine, Shihezi, Xinjiang China; 3 Department of Immunology, Shihezi University School of Medicine, Shihezi, Xinjiang China; 4 Department of Pathology, the First Affiliated Hospital, Shihezi University School of Medicine, Shihezi, Xinjiang China; 5 State Key Laboratory of Pharmaceutical Biotechnology, Chemistry and Biomedicine Innovation Center, Department of Biotechnology and Pharmaceutical Sciences, School of Life Sciences, Nanjing University, Nanjing, China

**Retraction Note: BMC Cancer (2023) 23:611**

10.1186/s12885-023-11086-9

The Editors have retracted this article. After publication, concerns were raised regarding highly similar images in Fig. 3b, where the siCtrl and siCirc#1 0 h images appear to overlap. The authors have stated that the two images were used by mistake and provided a revised figure for a correction. However, they have been unable to share the original images of alternative replicates of any in vitro experiments presented in this article.

The Editors therefore no longer have confidence in the presented data.

Lisha Zhou agrees to this retraction. Lianghai Wang does not agree to this retraction. Qianwen Wang, Jun Hou, Xiangwei Wu and Xueling Chen have not responded to any correspondence from the publisher about this retraction.

